# A Reverse Phase HPLC-UV and HPTLC Methods for Determination of Plumbagin in *Plumbago indica* and *Plumbago zeylanica*

**DOI:** 10.4103/0250-474X.49142

**Published:** 2008

**Authors:** K. P. Unnikrishnan, S. Sudhakar Raja, Indira Balachandran

**Affiliations:** Centre for Medicinal Plants Research, Arya Vaidya Sala, Kottakkal, Malappuram-676 503, India

**Keywords:** HPLC-UV, HPTLC, *Plumbago* spp.

## Abstract

A reverse phase HPLC method with UV detection has been developed and validated in order to quantify plumbagin, the bioactive marker of the roots of *P. indica* and *P. zeylanica*. A quantitative HPTLC method was also developed using hexane: ethyl acetate (8:2) as the mobile phase. The plumbagin content in the roots were determined using both the methods. *P. indica* was found to contain significantly higher amount of plumbagin than *P. zeylanica*. The HPLC and HPTLC methods described here are simple, rapid, accurate and sensitive.

*Plumbago indica* and *Plumbago zeylanica* (Plumbaginaceae) are the source for the well known ayurvedic drug ‘*Chitrakah*’. Both *P. indica* and *P. zeylanica* are widely distributed in tropics. From ancient time these species were known for their use in the treatment of leucoderma and other skin diseases including leprosy^1^. The root of the plant and its constituents are credited with potential therapeutic properties including antiatherogenic, cardiotonic, hepatoprotective and neuroprotective properties^2^. *P. zeylanica* is used in northern parts of India as the source of *Chitrakah*, while, *P. indica* is preferred by physicians in Kerala and other South Indian states. Pharmacological screening of various extracts of *Plumbago* species revealed that plumbagin, 2-ethyl-5-hydroxy-4-naphthoquinone (C_11_H_8_O_3_) is the bioactive compound responsible for the various medicinal activities of the plant^2^. At low doses, plumbagin has a stimulant action on central nervous system, muscles and secretion of sweat, bile and urine^3^. Antitumor and antifertility activities of plumbagin have been reported^4^. Interesting research activity is going on in the chemical front in making a number of plumbagin analogues by employing Schmid reaction, Thiel-Winter addition, allylic bromination and oxy-mercuration reactions^5^.

HPLC fingerprint analysis has now become a powerful tool for the quality control of raw plant materials. In comparison with HPLC, the greatest advantage of HPTLC procedure is that it does not require extensive clean up procedures of crude plant extracts even for quantitative analysis^6^. We describe here an analytical methodology for the HPLC estimation of plumbagin in *P. indica* and *P. zeylanica*. The results of HPLC analysis were compared with HPTLC and a relative assessment of the two chromatographic methods have been made. A HPTLC method for the quantification of plumbagin in different parts of *P. auriculata, P. rosea* and *P. zeylanica* and a normal phase liquid chromatographic method for the determination of plumbagin in *P. zeylanica* have been reported^7^,^8^. Another normal phase HPLC method for the separation of plumbagin from six different naturally occurring naphthaquinone using μ Bondapak CN column has been reported^9^. Subsequently, Stensen and Jensen used reverse phase liquid chromatography for the separation of plumbagin and its monomeric analogues^10^. To the best of our knowledge, there is no published reverse phase HPLC method for the quantification of Plumbagin in *P. indica* and *P. zeylanica*. More over a relative evaluation of both HPLC and HPTLC method is also done here. The method was optimized and validated so that it might be applied to assay biological samples.

Authenticated root samples of *P. indica* and *P. zeylanica* were collected from the Medicinal Plant Gene Pool Bank of CMPR, AVS, Kottakkal, India. Voucher specimens of all plant material employed in the study are preserved in the herbarium of Centre for Medicinal Plants Research, Kottakkal. Plant materials were shade dried and powdered.

A sample (1.0 g) of roots was kept in 10 ml ethanol for overnight. The extract was filtered and the solvent was evaporated off using rotary evaporator till dryness. The residue was redissolved in ethanol in order to obtain sample solution containing 100 μg/ml for analysis. Stock solutions of plumbagin (Sigma-Aldrich, Germany) were prepared in ethanol (analytical grade; Hayman Ltd, England) at 1000 μg/ml

A Shimadzu HPLC system (Kyoto, Japan) consisting of LC-10ATVP pump, a rheodyne injector, SPD M10AVP photodiode array detector and Class-VP 6.12 SP5 integration software was used for the analysis. The stationary phase was Phenomenex Luna C 18 (2) (250×4.6mm) column with 5 μ particle size with a C18 guard column (Phenomenex, 4×2.0 mm ID). The mobile phase consisting of methanol (HPLC grade, Merck) and sodium dihydrogen phosphate (5 mM) in the proportion (9:1 v/v) was used. The mobile phase was degassed by sonication before use. The column was equilibrated with the mobile phase for an hour and then pumped at the rate of 0.8 ml/min. For calibration, standard plumbagin solutions were prepared at concentration range of 1-20 μg/ml in ethanol. The standard solutions were injected in triplicate and the average detector responses measured. Plant samples were assayed in triplicate and detection was done at 265 nm.

The method was validated in terms of linearity, accuracy, interday and intra day precision. Accuracy of method was evaluated by carrying out recovery study. A known amount of plumbagin was added to 5 mg of previously analyzed powdered samples. These samples were extracted and analyzed by the procedure mentioned above. The plumbagin content was determined and the percentage recovery was calculated. We have assessed the precision of the HPLC analysis by processing aliquots of plumbagin standard through the procedure daily for a period of five days. The intraday precision was determined by analyzing standard plumbagin solutions in the concentration range of 5 to 10 μg/ml for three times on the same day.

Chromatographic analyses of the extracts were performed on silica gel 60 F 254 TLC plates (10×10 cm; Merck, Darmstadt, Germany). Samples were applied to the plates as sharp bands by means of Camag Linomat V sample applicator with a bandwidth of 6 mm. The distance between the tracks was 5 mm. Plates were developed in a TLC chamber previously saturated with the solvent system hexane:ethyl acetate (8:2). Development distance was 90 mm. Detection and quantification was performed with a Camag TLC Scanner 3 at 265 nm.

In order to check the repeatability of the method, samples in triplicate were extracted and analysed by maintaining the HPTLC conditions mentioned above. An aliquot of 100 μg/ml of standard solution was applied five times on TLC plates and were analysed to evaluate the reproducibility of the proposed method. Calibration graphs were recorded with sample amount ranging from 1-20 μg. (r = 0.991)

A reverse phase C-18 column with the mobile phase methanol and sodium dihydrogen phosphate (9:1 v/v) provided good separation of plumbagin from the herbal extracts (fig. 1). The detection wavelength was maintained at 265 nm as plumbagin showed good UV absorption in this region with no interference from the mobile phase or other components of the extract. The retention time of plumbagin was 4.21 min. The content of plumbagin in *P. indica* and *P. zeylanica* was found to be 0.2001 and 0.1601 %, respectively

**Fig. 1:**
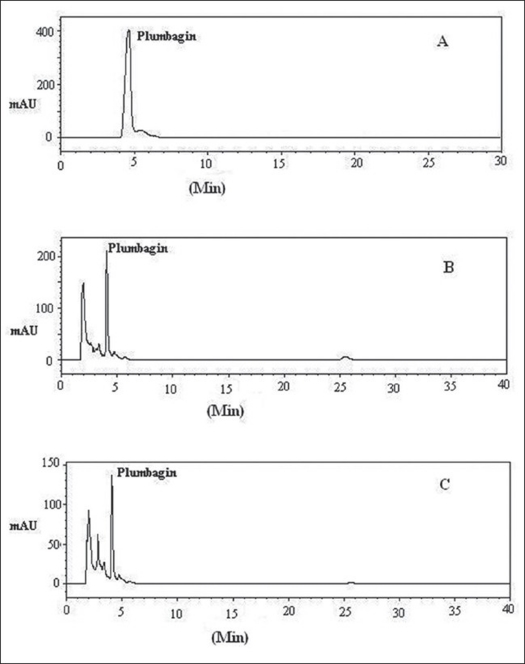
Typical HPLC chromatograms of plumbagin. Typical HPLC chromatograms for analysis of Plumbagin. (A) Plumbagin standard, (B) *P. indica*, (C) *P. zeylanica*

The regression analysis showed a linear relationship between concentration and the detector response. The linearity of response for plumbagin was found in the range 1-20 μg/ ml with a correlation coefficient of 0.9943. The HPLC analysis was found to be reproducible since the quantitative recovery of plumbagin (96.56±0.02% and 96.16±0.01%), was reliable throughout the range tested. The intraday and interday coefficient was found to be in the range of 0.9% to 3.2% and 0.30% to 4.1%. Lower values of intraday and inter day variation indicate that the method is precise.

With respect to HPTLC analysis, among the various solvent systems tried, the mobile phase that gave desired resolution with symmetrical and reproducible peaks was n-hexane: ethyl acetate (8:2). The use of this solvent system provides good separation of plumbagin with good resolution and separation from the other constituents of the plants. Using the proposed HPTLC method, Rf of plumbagin was determined as 0.67. The spectral characteristics of the peak at Rf 0.67 were also found to match, indicating that the compound corresponding to this Rf of the standard and test samples was identical. The chromatogram of standard plumbagin and that of test sample is shown in (fig. 2). The percentage of bioactive marker present in *P. indica* and *P. zeylanica* were determined to be 0.1921 and 0.1455%, respectively. The calibration curve was found to be linear in the range 1-20 μg.

**Fig. 2:**
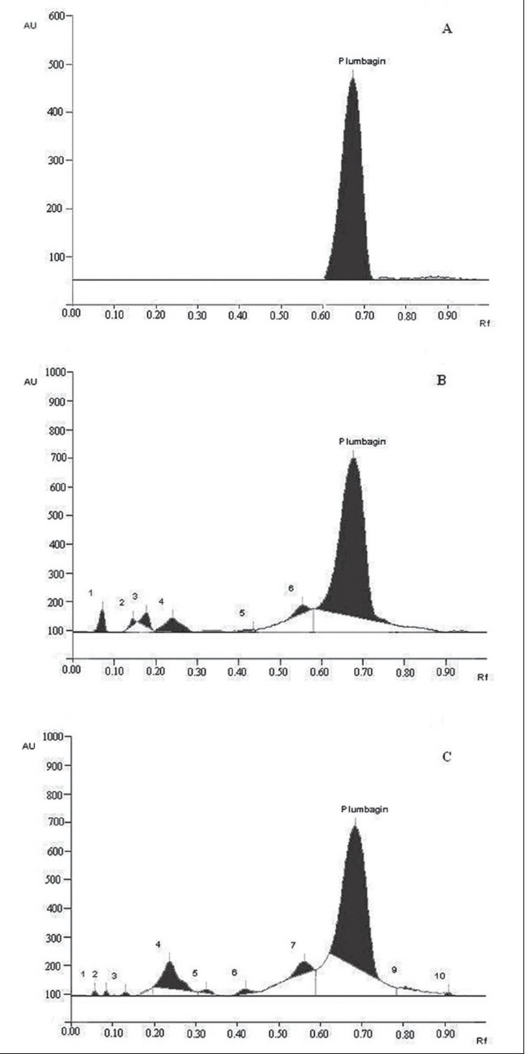
Typical HPTLC chromatograms of plumbagin. Typical HPTLC chromatograms for analysis of Plumbagin. (A) Plumbagin standard, (B) *P. indica*, (C) *P. zeylanica*

Estimation using both HPTLC and HPLC indicated that the plumbagin content is significantly high in the roots of *P. indica* compared to that of *P. zeylanica*. This explains the need for the purification process of *P. indica* being followed by ayurvedic people of South India^1^. The Ayurvedic Formulary of India also mentions the purification process of *P. indica*^11^. In our earlier work we have reported that purification process of *P. indica* results in the reduction of plumbagin content^12^. Plumbagin at higher doses is reported to be highly cytotoxic^13^.

Results indicate that HPTLC may be a method of choice for routine fingerprint analysis due to the possibility of simultaneously analyzing several samples in less time than by HPLC. As for quantification HPLC seems to be more sensitive and precise than HPTLC, which involves fluorescence detection with densitometry. The intra day reproducibility value of 4.1% obtained for HPLC analysis also indicates the reliability of the HPLC method in quantification of plumbagin.
